# Correlation between mismatch repair statuses and the prognosis of stage I–IV colorectal cancer

**DOI:** 10.3389/fonc.2023.1278398

**Published:** 2024-01-29

**Authors:** Guojun Tong, Guiyang Zhang, Yan Hu, Xuting Xu, Yanyan Wang

**Affiliations:** ^1^ Colorectal Surgery, Huzhou Central Hospital, The Affiliated Huzhou Central Hospital of Huzhou University, Zhejiang, China; ^2^ Central Laboratory, Huzhou Central Hospital, The Affiliated Huzhou Central Hopsital of Huzhou University, Zhejiang, China; ^3^ Department of English, Huzhou Vocational and Technical College, Zhejiang, China; ^4^ Pathology Department, Huzhou Central Hospital, The Affiliated Central Hospital of Huzhou University, Zhejiang, China

**Keywords:** CRC, Prognosis, MSI, OS, DFS

## Abstract

**Background:**

The role of microsatellite instability (MSI) and prognosis for stage II–III colorectal cancer (CRC) has been described, but the role of MSI in stage I and IV CRC is controversial.

**Methods:**

A total of 2,540 CRC patients were collected from Huzhou Central Hospital, China, from January 2006 to 2016, and 783 cases were excluded. This retrospective study illustrates the correlation between MMR status and prognosis for 1,757 CRC patients as well as the correlation between MSI and prognosis for CRC patients. Two groups were classified as MSI-H and MSI-L&MSS. If the expression of one or more mismatch repair (MMR) proteins was negative, it was considered as microsatellite instability high expression (MSI-H), whereas positive expression was considered as microsatellite instability low expression and microsatellite stability (MSI-L&MSS), as assessed by correlation analyses. Overall and disease-free survival were analyzed using the Kaplan–Meier method. Univariable and multivariable analyses were conducted using Cox regression.

**Results:**

Preoperative serum S-CEA, positive lymph, tumor size, pathologic tumor (Pt) status, node (N) stage, differentiation, chemotherapy, and the 8th Edition of the American Joint Committee on Cancer (AJCC-8) were significantly correlated with MSI (P=0.028, 0.037, 0.019, 0.007, 0.002, <0.001, <0.001, and <0.001, respectively), whereas tumor location was not associated with MSI. Univariable and multivariable analyses showed that MSI was an independent factor for CRC. The 5-year overall survival (OS) and 5-year disease-free survival (DFS, P<0.001) rates differed significantly between the two groups in stages II, III, and IV, whereas stage I did not show a significant difference (P>0.05).

**Conclusion:**

MSI-H was associated with a good prognosis for stages II to IV, whereas stage I did not show any significant correlation. Moreover, MSI expression was an independent prognostic factor.

## 1 Introduction

CRC is one of the most common malignancies in Western countries, and its incidence is continually increasing in Asian countries (1). Our previous studies showed that the expression of several genes, such as *CEA* and proliferation marker protein Ki-67 (*Ki67*), were associated with CRC prognosis (2, 3). Genomic instability is one of the main characteristics of CRC, including MSI, chromosome instability, and CpG island methylation (CIMP). A microsatellite is a simple repetitive sequence with high mutations in the genome. The phenomenon of microsatellite changes during DNA replication is termed MSI. The lack of mismatch repair (MMR) protein (d-MMR) due to the mutation or abnormal expression of the MMR genes (including *MLH1*, *MSH2*, *MSH6*, and *PMS2*) underlie MSI. According to the number of mutation sites, the MSI can be divided into high MSI (MSI-H), low MSI (MSI-L), and no MSS type. Among these, MSI-L is classified due to its clinical characteristics, which are very similar to MSS, indicating a similar tumor location, differentiation, lymph metastasis, and pathological staining attributed to parallel gene expression (4).

Nonetheless, the prognostic role of MSI in CRC is controversial. Sargent DJ et al. (5) did not find any significant correlation between MSI status and prognosis. Recently, Taieb et al. (6) conducted a summary of seven studies and multivariate analysis in 2,630 patients with CRC recurrence. Patients with the MSI-H/d-MMR phenotype showed improved prognosis in the pre-immunotherapy stage after adjuvant chemotherapy. A meta-analysis of 3,063 patients with metastatic CRC by Venderbosch et al. (7) showed that patients with MSI-H/d-MMR had lower progression-free survival (PFS) and OS, which rendered the evidence of the short survival of phase IV MSI-H sufficient. Another study provided an opposite theory that MSI-H had a good prognosis when the ratio of the sample of early and late patients was 1:1 (8). A recent study (9) has shown a dramatical improvement in survival with immunotherapy (programmed death-ligand 1 [PD-(L)1] cytotoxic T-lymphocyte-associated antigen 4 [CTLA-4] blockage) in metastatic or non-metastatic MSI/dMMR CRC and reported new treatment recommendations for this unique CRC population. Despite their efficacy, primary and secondary resistance to immune checkpoint inhibitors (ICIs) are observed in more than 50% of MSI-H/dMMR CRC patients, and in the future, how to identify these patients and overcome resistance will be an important challenge. In 2017, the FDA approved two ICIs (pembrolizumab and nivolumab) for the treatment of MSI-H/dMMR metastatic CRC (mCRC). In 2018, the CheckMate-142 trial demonstrated the successful treatment of mCRC based on “double immunity” provided by nivolumab with ipilimumab, a regimen that may become a standard first-line treatment for MSI-H mCRC. In 2018, the FDA approved nivolumab alone or with ipilimumab for patients who progressed to MSI-H mCRC after standard chemotherapy (10). Colorectal cancer (CRC) patients, especially those with deficient mismatch repair (dMMR)/microsatellite instability-high (MSI-H) tumors, whose sensitivity to immune checkpoint inhibitors (ICIs) is significantly higher than that of patients with microsatellite-stable (MSS)/microsatellite instability-low (MSI-L) tumors, have derived clinical benefits from immunotherapy (10). Lenz et al. (11) recently showed that nivolumab plus low-dose ipilimumab provide a robust and durable clinical benefit and was well tolerated as a first-line treatment for MSI-H/dMMR mCRC. Based on these promising data, randomized studies are warranted. MSI-H has been speculated to have a better prognosis in patients with stage I and II CRC, but its role in patients with stage III and IV CRC is still controversial (8). Through a recent literature search, we found MSI-H/dMMR could guide treatments of advanced or metastatic CRC, but there is still a lot of controversy about the effect of MSI status on the prognosis of conventional treatment for stage I-IV CRC, which was one purpose of our article. Therefore, the current study aimed to explore the correlation between MSI status and the prognosis of stage I–IV CRC.

## 2 Materials and methods

### 2.1 Clinical data

**Figure 1:**
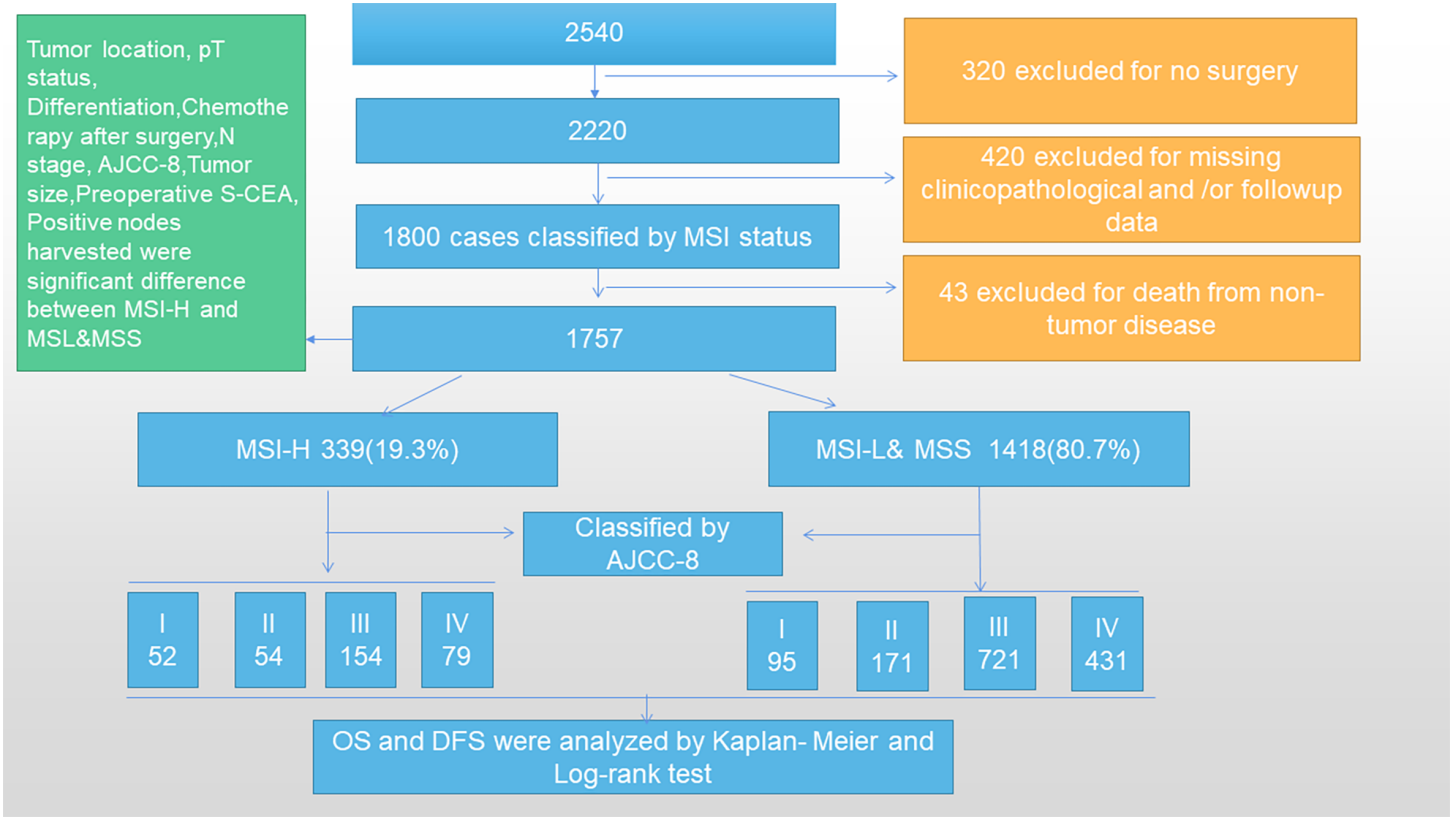
Study flow according to the inclusion and exclusion criteria.

A total of 2,540 CRC patients were collected in the Colorectal Surgery Department of Huzhou Central Hospital, China from January 2006 to 2016. Subsequently, 320 cases were excluded for no surgery and 420 cases were excluded for missing general clinicopathological and/or follow-up data. A total of 1,800 cases were classified by MSI status and 43 cases were excluded due to death from non-tumor disease during follow-up. Finally, 1,757 cases with stage I–IV CRC were included in our study and divided into two groups according to MSI status. The inclusion criteria were as follows: patients diagnosed with CRC through colonoscopy, computed tomography (CT), and pathological tests inside or outside our hospital; no preoperative adjuvant treatment; surgery in our department; normal lymph node dissection indicating that ≥12 lymph nodes were detected, although a small number of samples were included in this article, and only 10–11 lymph nodes were detected; CRC-related death as a termination event; postoperative routine immunohistochemical (IHC) analysis and pathological examination for MSI (*MLH1*, *MSH6*, *MSH2*, and *PMS2* gene expression); and postoperative chemotherapy determined by AJCC-8 guidelines. The exclusion criteria were as follows: serious heart, brain, liver, and lung diseases that did not require surgery; non-CRC factors leading to patient death; and follow-up data missing and/or clinicopathological data missing (**Figure 1**). Based on the exclusion and inclusion criteria, we selected cases that would minimize the bias.

### 2.2 Follow-up

The patients were routinely followed up in the outpatient clinic 2 weeks postoperatively and every 3 months for the first year, then every 6 months for the second year, and every year for the next 3 years up to 5 years after the operation. The follow-up data were collected by phone calls and outpatient records.

### 2.3 MSI status classification

**Figure 2:**
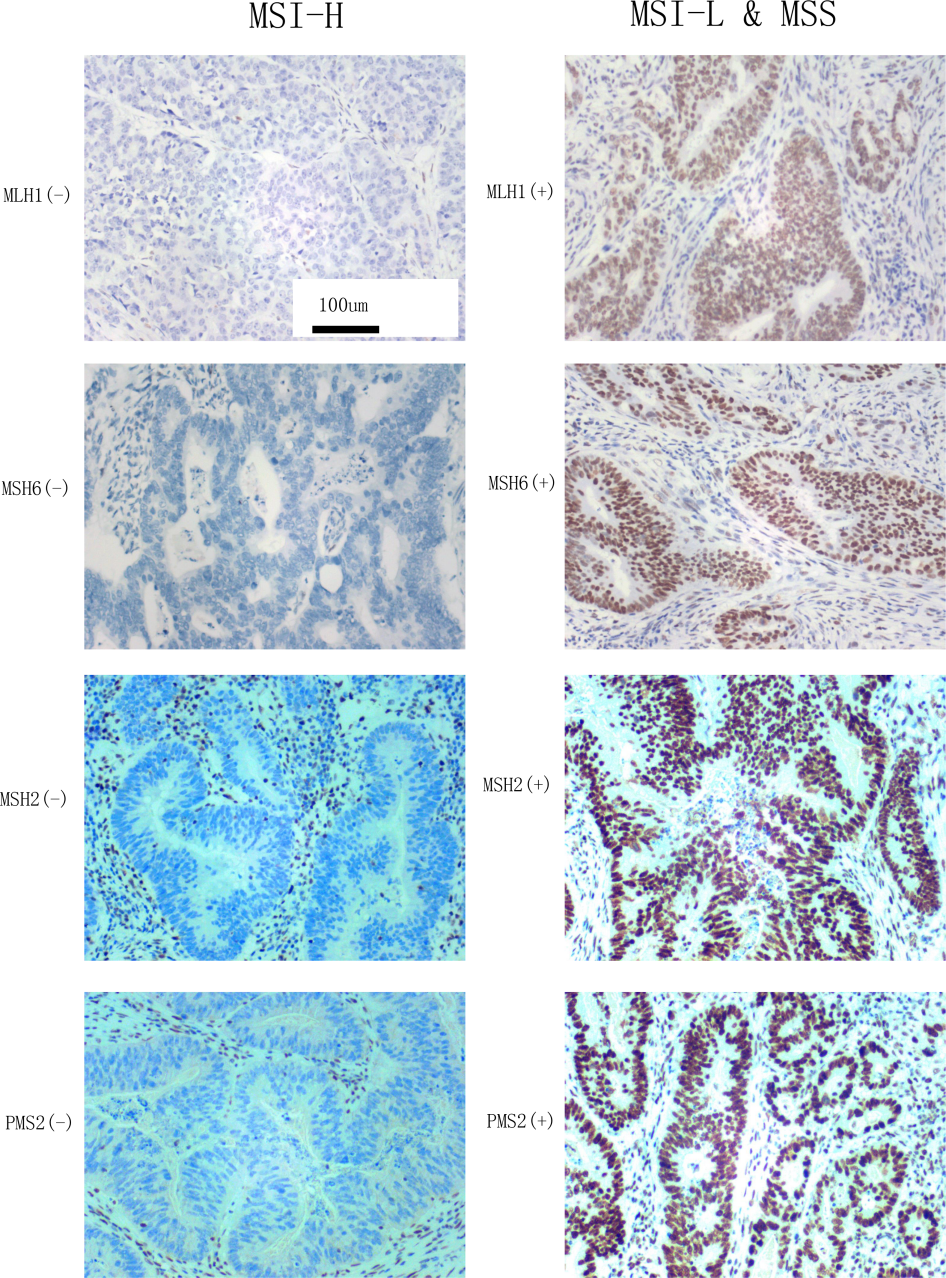
MLH1, MSH6, MSH2, and PMS2 expression observed by microscopy (×200) and the classification of MSI status according to reference 8. Left: the negative expression of MLH1, MSH6, MSH2, and PMS2 indicate MSI-H. Right: The positive expression of MLH1, MSH6, MSH2, and PMS2 indicate MSI-L&MSS.

The d-MMR caused by mutation or abnormal expression of MMR genes (including *MLH1*, *MSH2*, *MSH6*, and *PMS2*) is the main cause of MSI. According to the number of mutation sites, MSI can be divided into MSI-H, MSI-L, and MSS. Among these, MSI-L is often classified as a class because of its clinical characteristics, which are highly similar to MSS (4). If more than one of or only one of MLH1, PMS2, MSH2, and MSH6 antibodies was negative, the MMR protein expression was considered lacking and referred to as d-MMR, characterized by MSI-H. Subjects with positive expression of all four antibodies were judged to have a normal expression of MMR protein, referred to as PMMR, which indicated MSI-L or MSS (12). Based on these criteria, the MSI status was classified as MSI-H and MSI-L&MSS (**Figure 2**).

### 2.4 MSI detection by elivision immunohistochemistry

The MMR protein antibodies *MLH1, PMS2, MSH2, and MSH6* were purchased from the Fuzhou Maixin company (Innovation, Fuzhou, China). The secondary antibody and chromogenic system, Dako k8002 system reagent and a Dakao link48 automatic immunohistochemical machine, respectively, were used to evaluate the IHC staining. The four antibodies were used for nuclear staining. They were judged as positive when there was tumor nuclear staining and negative when there was no tumor nuclear staining. The results of IHC staining were interpreted by two pathologists with the title of Chief Physician or above. The interpretation criteria were as follows: the protein expression of *PMS2, MSH2, MSH6*, and *MLH1* were detected in the nucleus as brownish-yellow particles. According to these judgment criteria (9), we selected the cell staining of *MLH1*, *MSH6*, *MSH2* and *PMS2* to classify the MSI which were stained in our pathological department (**Figure 2**).

### 2.5 Statistical analysis

SPSS 21.0 (SPSS Inc., IL, USA) was used to input all clinical and follow-up data. The clinicopathological measurement data in groups with MSI-H and MSI-L&MSS expression profiles were compared using single factor analysis of variance, whereas counting data were analyzed using Pearson’s chi-square test. The study parameters were defined as the time from operation to death or from operation to 5 years after the operation, which were considered as the study cutoff points. Kaplan–Meier and log-rank tests were used to analyze the 5-year OS and DFS rates of CRC according to MSI expression. Cox regression for univariate and multivariate analysis is based on different clinical, pathological, and biochemical variables. In multivariate analysis, all variants in the first index were considered as a reference (input). P<0.05 (two-sided) indicated a statistically significant difference.

## 3 Results

### 3.1 General data

The MSI-H group consisted of 339 cases (19.3%), 171 (9.7%) males and 168 (9.6%) females. The MSI-L&MSS group consisted of 1,418 (80.7%) cases, among which 717 (40.8%) were males and 701 (39.9%) were females. A comparison of sex between the two groups did not show any significant difference (P=0.968). The mean ages of the MSI-H and MSI-L&MSS subgroups were 63.9 ± 13.9 and 63.6 ± 13.9 years, which did not differ significantly (P=0.725). The data are summarized in **Figure 1** and **Supplementary Table 1**.

### 3.2 Clinicopathological features

Some clinicopathological parameters showed no significant differences in the American Society of Anesthesiologists (ASA) score (P=0.794), complications (n, P=0.742), operation time (min, P=0.812), resection length (cm, P=0.194), blood loss (mL, P=0.546), lymph harvest (n, P=0.662), and operation method (n, P=0.151) between the two groups. The comparisons of significance for several parameters revealed the following: preoperative S-CEA (P=0.027), tumor location (P=0.006), Pt status (P<0.001), differentiation (P<0.001), postoperative chemotherapy (P<0.001), N stage (P=0.003), AJCC-8 (P<0.001), tumor size (P=0.040), and positive lymph (P=0.041). The details of mean, number, percentage, and comparisons are summarized in **Supplementary Table 1**.

### 3.3 Correlation analyses between MSI and clinicopathological features

Nine statistically significant variables in both groups were included in the bivariate correlation analysis. The results showed that there is no correlation between MSI and tumor location (Spearman’s rho=-0.022, P=0.355), whereas the other eight variations had significant correlations with MSI (all P<0.05). Spearman’s rho and P-values are shown in **Supplementary Table 2**.

### 3.4 Comparisons of 5-year OS and DFS rates between MSI-H and MSI-L&MSS according to AJCC-8 stratification

**Figure 3:**
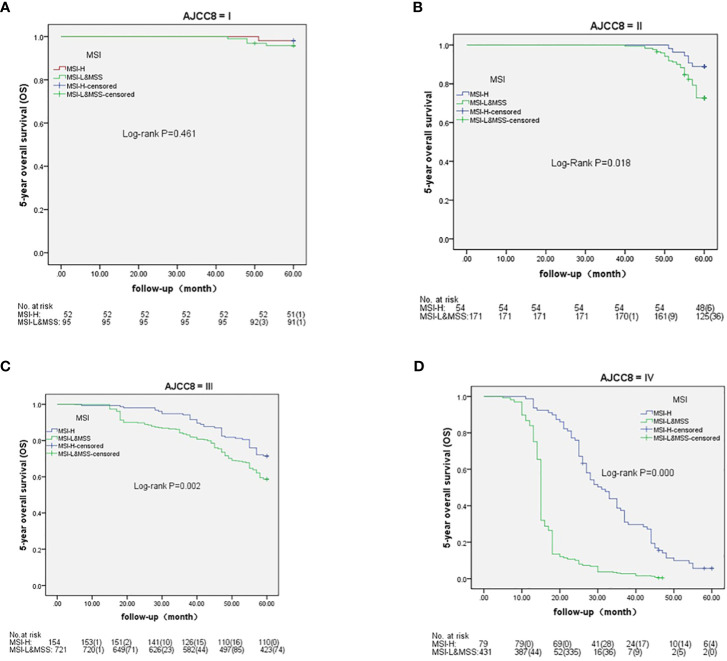
OS analysis by Kaplan–Meier and log-rank tests in AJCC-8 stratification with number table at risk of CRC (I-IV stages) between MSI-H and MSI-L&MSS groups. **(A)**: In stage I ,there is no significance (P= 0.461) between MSI-H and MSI-L&MSS groups; **(B)**: In stage II there is significance between the two groups (P=0.018); **(C)**: In stage III there is significance between the two groups (P=0.002); **(D)**: In stage IV there is significant difference between the two groups(P<0.001).

**Figure 4:**
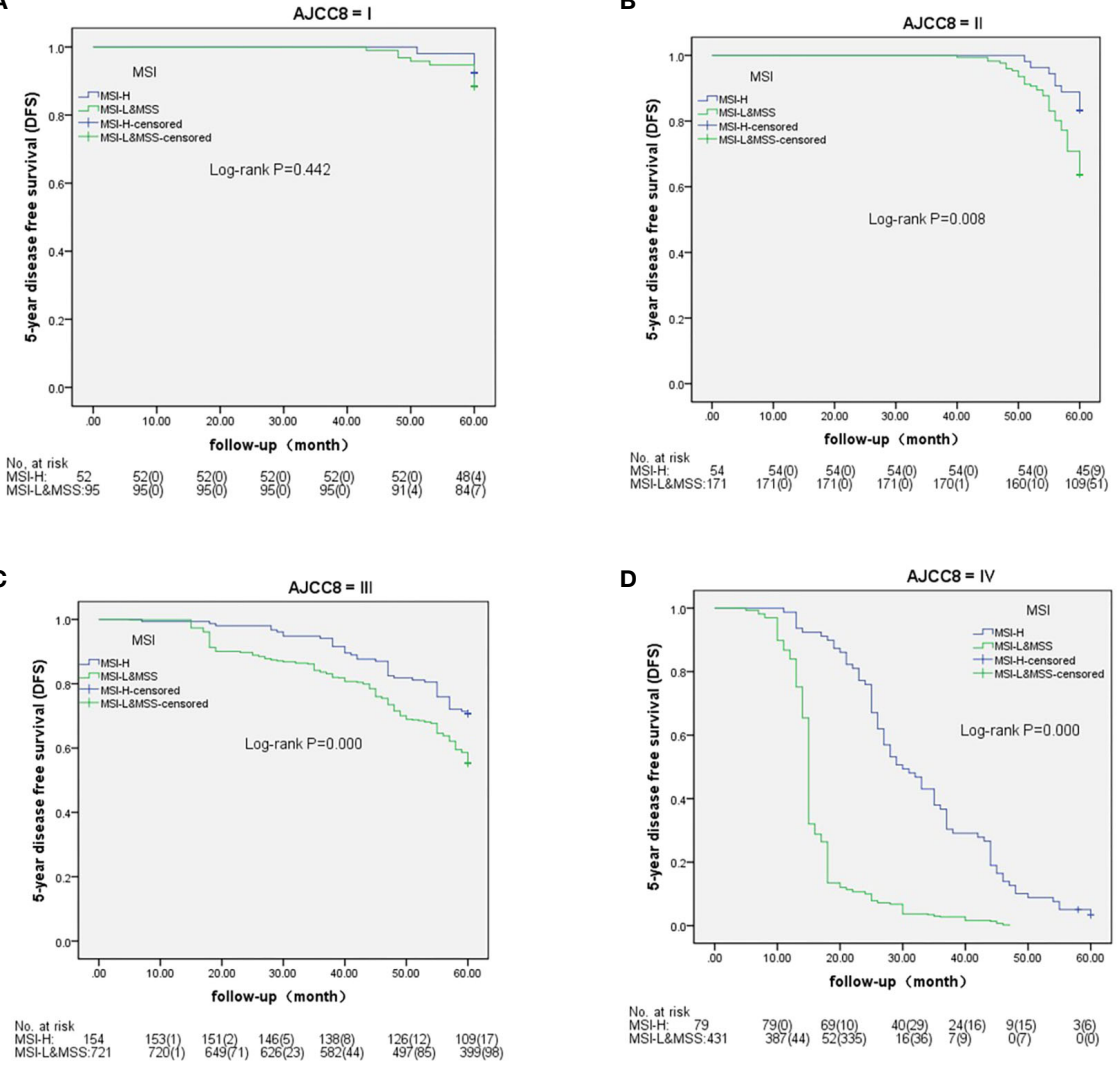
DFS analysis by Kaplan–Meier and log-rank tests in AJCC-8 stratification with number table at risk of CRC (I-IV stages) between MSI-H and MSI-L&MSS groups. **(A)**: In stage I ,there is no significance (P=0.442) between MSI-H and MSI-L&MSS groups; **(B)**: In stage II there is significance between the two groups (P=0.008); **(C)**: In stage III there is significance between the two groups (P<0.001); **(D)**: In stage IV there is significant difference between the two groups (P<0.001).

**Figure 5:**
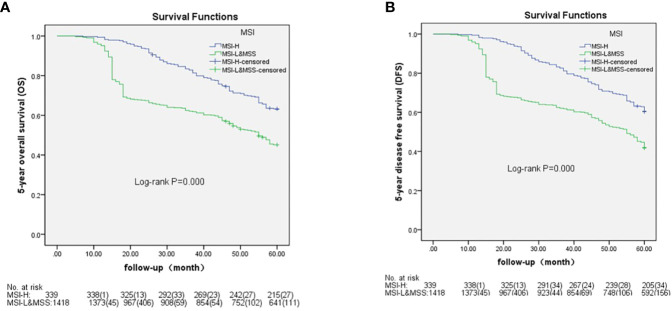
OS ad DFS analysis by Kaplan–Meier and log-rank tests out AJCC-8 stratification with number table at risk of CRC between MSI-H and MSI-L&MSS groups. **(A)**: There is significance between the two groups (P<0.001); **(B)**: There is significant difference between the two groups (P<0.001).

Based on AJCC-8, the 5-year OS in stages I to IV was 98.1%, 88.9%, 71.4%, and 5.6% in MSH-H and 95.8%, 72.7%, 58.7%, and 0.5% in MSI-L&MSS, respectively. The results of the log-rank test showed that there were no significant differences in stage I (P=0.461), whereas stages II to IV showed significant differences (P=0.018, 0.002, and <0.001, respectively) (**Figure 3**). Based on AJCC-8, DFS was 92.3%, 83.3%, 70.8%, and 3.4% for stages I to IV in MSI-H and 88.4%, 63.7%, 55.3%, and 0.2% in MSI-L&MSS, respectively. The log-rank test results showed that there was no significant difference in stage I (P=0.442), whereas significant differences were noted in stages II to IV (P=0.008, others P<0.001; **Figure 4**). Based on the stratification analysis, the 5-year OS and DFS were 63.3% and 60.5 for MSI-H and 45.1% and 41.7% for MSI-L&MSS, respectively, indicating statistical significance (all P<0.001, **Figure 5**).

### 3.5 Univariate and multivariate analysis of CRC prognosis

The univariate analyses of the number of cases, hazard ratio (HR), 95% confidence interval (CI) for survival time, 5-year OS, and P-value were assessed on gender, age, tumor size, Pt status, differentiation, N stage, AJCC-8, and MSI status, and the results showed that gender had no significant difference (P=0.303) but other parameters differed significantly between the two groups (all P<0.001). The details are shown in **Supplementary Table 3**. The parameters with significant differences in the univariate analyses were analyzed by Cox regression and multivariate analysis. The outcomes showed that tumor size, Pt status, differentiation, N stage, AJCC-8, and MSI are independent factors for CRC prognosis (P=0.044, all other P<0.001). The analysis of out and in stratification is shown in **Supplementary Table 3**.

## 4 Discussion

MSI is characterized by short sequence repeats (SSRs) or short tandem repeats (STRs) of repeated DNA sequences of various lengths (13). Microsatellites are widely distributed throughout the genome in a non-random manner and are prone to mutations during DNA replication (14). The MMR system consists of a family of enzymes that detect DNA replication errors (such as mismatches between the two strands of DNA). The MMR system includes the *MHL1*, *MSH2*, *MSH6*, and *PMS2* genes. MSI has been frequently observed in various malignancies and has become a new research hotspot (15). It is associated with Lynch syndrome, which develops CRC easily (16). Although the prognostic role of MSI-H has been elaborated, many prognostic studies worldwide have been inconclusive (14). Studies have confirmed that MSI tumors have a better prognosis than microsatellite stable CRC, but MSI cancers do not necessarily have the same response to the chemotherapeutic strategies used to treat microsatellite stable tumors. Specifically, stage II MSI tumors might not benefit from 5-fluorouracil-based adjuvant chemotherapy regimens. New data suggested possible advantages of irinotecan-based regimens, but these findings require further clarification (17). Colorectal cancers with MSI have distinctive features, including a tendency to arise in the proximal colon, poor differentiation, lymphocytic infiltration, and mucinous or signet-ring histology. Patients with MSI tumors appear to have a better prognosis than those with microsatellite stable tumors, but curiously the responses to 5-fluorouracil-based chemotherapy regimens are poorer with MSI tumors. Preliminary data suggested possible advantages of irinotecan-based regimens, but these findings need validation in well-designed clinical trials (18).The above conclusions did not classify MSI status and the results were illegible. Zoran Gatalica et al. (19) showed that heterogeneous MSI-H colorectal carcinomas as a group showed some distinct biological characteristics when compared with CRC with stable or low-level microsatellite instability. In their present review, they highlighted therapeutically relevant characteristics of MSI-H tumors that could lead to specific responses to some conventional chemotherapy or novel targeted therapy agents. With a similar viewpoint to E. Vilar’s (17), F. Battaglin, M et al. (20) considered that SI-high (MSI-H) status was associated with a better prognosis in early-stage CRC but a lack of benefit from adjuvant treatment with 5-fluorouracil in stage II disease. Moreover, MSI has emerged as a predictor of sensitivity to immunotherapy-based treatments. Based on the above studies, we believe that the role of MSI status in the prognosis of stage I–IV CRC remains unclear. Therefore, the present study aimed to explore the correlation between MSI expression and CRC prognosis from stages I to IV.

This study revealed that MSI-H usually occurs in the left colon and rectum, which was inconsistent with the previous findings that indicated that MSI-H occurs in the right colon (21). However, the present study suggests that MSI-L&MSS is likely to occur in the left colon and rectum for unknown reasons. The operation methods did not differ significantly between the MSI-H and MSI-L&MSS groups. The results also showed that MSI-H had weak tumor invasion and lymph node metastasis. A. Perrier et al. (22) revealed that MSI status exhibits a high response rate in almost all cancer types, and MSI-L was invasive and had lymph node metastasis, which was consistent with our study. Previous studies (23, 24) have shown that MSI-L&MSS has poor tumor differentiation, an elevated signet ring appearance, poor tumor differentiation, and a large tumor. Smyth et al. (25) showed that MSI-L&MSS had young age (median, 62 years) while MSI-H comprised older elderly (median, 66 years), which were not consistent with our study that did not show a significant difference between the two groups (P=0.725, **Supplementary Table 1**); additionally, bivariate correlation analysis did not establish any correlation between age and MSI status. Older CRC patients had a better prognosis than younger patients because tumor cell division was slower in older patients (26–28). The efficacy of PD-1 blockade compared with chemotherapy as a first-line therapy for MSI-H-dMMR advanced or metastatic colorectal cancer is unknown (29). In our study, MSI-H had a smaller chemotherapy proportion but a better prognosis due to its adequate biological behavior compared with MSI-L&MSS. The bivariate correlation analysis revealed that variations in preoperative S-CEA, positive lymph number, tumor size, tumor invasion (Pt status), N stage, differentiation, chemotherapy, and AJCC-8 were correlated to MSI status but not tumor location.

Several retrospective studies, including a systematic review and a meta-analysis, support the favorable stage-adjusted prognosis of MSI-H compared with MSS CRC patients (30–33). Typically, MSI-H has a good prognosis in early CRC. However, a literature search retrieved a few studies on the prognostic analysis of MSI-H in stage I CRC. Reportedly, MSI-H had a good prognosis in stage I–III CRC through 5-FU chemotherapy (34). Recent studies have shown that immunotherapy for stage IV CRC has a significant positive prognosis for MSI-H expression (35–37). However, only a few comparative studies are available on the prognosis of MSI-H CRC patients compared with MSI-L&MSS patients in stage IV. Toh et al. (38) assessed stage IV CRC without immunotherapy and found that MSI-H was not associated with any survival benefit, suggesting that a survival benefit was evident in both stage II and III CRC, and MSI-H was neither a robust prognostic marker in stage I nor stage IV CRC without immunotherapy. Moreover, in the current study, no significant prognosis was detected in stage I, which was consistent with the report by James et al. Interestingly, MSI-H had a better prognosis than MSI-L&MSS in stage IV accompanied by the therapy according to the National Comprehensive Cancer Network (NCCN) guidelines. The reasons for this might be that MSI-H is related to the biological behavior of the tumors, such as better tumor differentiation, poor tumor infiltration ability, and a low positive rate of lymph node metastasis, which were consistent with previous findings (39). G3 CRC was significantly correlated with high MSI (MSI-H) compared with G1 and G2 (P=0.002; odds ratio, 5.750), which improved the prognosis of MSI-H (40). Multivariate analysis identified MSI-H as an independent prognosis factor for CRC patients, and the results were consistent with the published literature (41, 42).

Nevertheless, the current study has some limitations. This is a single-center clinical study. The selected cases were collected up to January 2016, and hence, the CRC patients experiencing the optimal effect of MSI-H immunotherapy are not included. The MSI status tested by immunohistochemistry may have a small bias compared with other testing methods. Moreover, as *KRAS* gene testing was carried out relatively late in this unit, the relationship between MIS status and KRAS mutations and their prognosis with colorectal cancer were not studied. Aspects of immune parameters (humoral and cell-mediated), or the mutational statuses of genes such as *Ras* and *Raf* at various stages and correlated with MMR statuses, were not investigated in this study due to the immature technology. These are other limitations in the study. Prospective studies with different chemotherapy treatments, MSI statuses, CRC prognoses, and other tumor marks associated with CRC should be performed in future if possible.

## 5 Conclusions

MSI-H was associated with a good prognosis for stage II–IV CRC patients, but no significance was detected for stage I. Moreover, MSI expression was an independent factor for CRC patients. Further studies regarding the correlations between MSI status and stage I–IV CRC from multicentral institutions should appear. Prognosis using immunotherapy and chemotherapy based on MSI status guidance for advanced colorectal cancer is the next step in our research.

## Data availability statement

The original contributions presented in the study are included in the article/**Supplementary Material**. Further inquiries can be directed to the corresponding author.

## Ethics statement

The studies involving humans were approved by the ethics committee of Huzhou Central Hospital (No. 202110015-01). The studies were conducted in accordance with the local legislation and institutional requirements. Written informed consent for participation in this study was provided by the participants’ legal guardians/next of kin.

## Author contributions

GT: Conceptualization, Data curation, Formal analysis, Funding acquisition, Investigation, Methodology, Supervision, Validation, Writing – original draft, Writing – review & editing. GZ: Conceptualization, Methodology, Supervision, Writing – original draft, Writing – review & editing. YH: Writing – original draft, Writing – review & editing. XX: Conceptualization, Methodology, Supervision, Writing – original draft, Writing – review & editing. YW: Data curation, Validation, Writing – original draft, Writing – review & editing.

## References

[1] Tong G Zhang G Liu J Zheng Z Chen Y Cui E. A meta-analysis of short-term outcome of laparoscopic surgery versus conventional open surgery on colorectal carcinoma. Medicine (2017) 96:e8957–7. 10.1097/MD.0000000000008957 PMC5728795 29310394

[2] Tong G Zhang G Liu J Zheng Z Chen Y Niu P. Cutoff of 25% for Ki67 expression is a good classification tool for prognosis in colorectal cancer in the AJCC−8 stratification. Oncology Reports (2020) 43:1187–98. 10.3892/or.2020.7511 PMC7058009 32323802

[3] Tong G Xu W Zhang G Liu J Zheng Z Chen Y. The role of tissue and serum carcinoembryonic antigen in stages I to III of colorectal cancer-A retrospective cohort study. Cancer Med (2018) 7:5327–38. 10.1002/cam4.1814 PMC6246925 30302946

[4] Cheng Yu PH Shao J Hu K. Progress on Microsatellite Instability in the prognosis and Treatment of Colorectal Cancer. J Cancer Control Treat (2020) 33(4):364–9.

[5] Sargent DJ Shi Q Yothers G Tejpar S Bertagnolli MM Thibodeau SN. Prognostic impact of deficient mismatch repair ( dMMR) in 7, 803 stage II / III colon cancer ( CC) patients ( pts) :A pooled individual pt data analysis of 17 adjuvant trials in the ACCENT database. J Clin Oncol (2014) 32(5):3507.

[6] Taieb J Shi Q Pederson L Alberts S Wolmark N Van Cutsem E. Prognosis of microsatellite instability and/or mismatch repair deficiency stage III colon cancer patients after disease recurrence following adjuvant treatment: results of an ACCENT pooled analysis of seven studies. Ann Oncol (2019) 30:1466–71. doi: 10.1093/annonc/mdz208 PMC7360150 31268130

[7] Venderbosch S Nagtegaal ID Maughan TS Smith CG Cheadle JP Fisher D. Mismatch repair status and BRAF mutation status in metastatic colorectal cancer patients: a pooled analysis of. CAIRO2 COIN Focus studies Clin Cancer (2014) 20:5322–30. 10.1158/1078-0432.CCR-14-0332 PMC4201568 25139339

[8] Liu C Ji Le Tiecheng B. Analysis and Reflection on the correlation between MSI status and prognosis and clinicopathological features of patients with stage II and IV colon cancer. Chin J Gen Surg (Electronic Edition) (2019) 13(3):283–6.

[9] Taieb J Svrcek M Cohen R Basile D Tougeron D Phelip JM. Deficient mismatch repair/microsatellite unstable colorectal cancer: Diagnosis, prognosis and treatment. Eur J Cancer (Oxford Engl 1990) (2022) 175:136–57. doi: 10.1016/j.ejca.2022.07.020 36115290

[10] Zhang X Wu T Cai X Dong J Xia C Zhou Y. Neoadjuvant Immunotherapy for MSI-H/dMMR Locally Advanced Colorectal Cancer: New Strategies and Unveiled Opportunities. Front Immunol (2022) 13:795972. doi: 10.3389/fimmu.2022.795972 35371084 PMC8968082

[11] Lenz HJ Van Cutsem E Luisa Limon M Wong KYM Hendlisz A Aglietta M. First-Line Nivolumab Plus Low-Dose Ipilimumab for Microsatellite Instability-High/Mismatch Repair-Deficient Metastatic Colorectal Cancer: The Phase II CheckMate 142 Study. J Clin Oncol (2022) 40:161–70. doi: 10.1200/JCO.21.01015 34637336

[12] Li X Liu H Liang M Chen H Liang L. Clinicopathological features and types of microsatellite instability in 1394 patients with colorectal cancer. Nan Fang Yi Ke Da Xue Xue Bao (2020) 40:1645–50. 10.12122/j.issn.1673-4254.2020.11.17 PMC7704367 33243738

[13] Gemayel R Cho J Boeynaems S Verstrepen KJ. Beyond junk-variable tandem repeats as facilitators of rapid evolution of regulatory and coding sequences. Genes (2012) 3:461–80. doi: 10.3390/genes3030461 PMC3899988 24704980

[14] Jin Z Sinicrope FA. Prognostic and Predictive Values of Mismatch Repair Deficiency in Non-Metastatic Colorectal Cancer. Cancers (Basel) (2021) 13:300. doi: 10.3390/cancers13020300 33467526 PMC7830023

[15] Yang G Zheng R-Y Jin Z-S. Correlations between microsatellite instability and the biological behaviour of tumours. J Cancer Res Clin Oncol (2019) 145:2891–9. doi: 10.1007/s00432-019-03053-4 PMC6861542 31617076

[16] Dabir PD Bruggeling CE van der Post RS Dutilh BE Hoogerbrugge N Ligtenberg MJL. Microsatellite instability screening in colorectal adenomas to detect Lynch syndrome patients? A systematic review and meta-analysis. Eur J Hum Genet (2020) 28:277–86. doi: 10.1038/s41431-019-0538-7 PMC7028913 31695176

[17] Vilar E Gruber SB. Microsatellite instability in colorectal cancer-the stable evidence, Nature reviews. Clin Oncol (2010) 7:153–62. 10.1038/nrclinonc.2009.237 PMC3427139 20142816

[18] Pino MS Chung DC. Microsatellite instability in the management of colorectal cancer. Expert Rev Gastroenterol Hepatol (2011) 5:385–99. doi: 10.1586/egh.11.25 21651356

[19] Gatalica Z Vranic S Xiu J Swensen J Reddy S. High microsatellite instability (MSI-H) colorectal carcinoma: a brief review of predictive biomarkers in the era of personalized medicine. Familial Cancer (2016) 15:405–12. doi: 10.1007/s10689-016-9884-6 PMC4901118 26875156

[20] Battaglin F Naseem M Lenz HJ Salem ME. Microsatellite instability in colorectal cancer: overview of its clinical significance and novel perspectives. Clin Adv Hematol Oncol (2018) 16:735–45. PMC7493692 30543589

[21] Ghidini M Petrelli F Tomasello G. Right Versus Left Colon Cancer: Resectable and Metastatic Disease. Curr Treat options Oncol (2018) 19:31. doi: 10.1007/s11864-018-0544-y 29796712

[22] Perrier A Didelot A Laurent-Puig P Blons H Garinet S. Epigenetic Mechanisms of Resistance to Immune Checkpoint Inhibitors, Biomolecules, 10. (2020). doi: 10.3390/biom10071061 PMC7407667 32708698

[23] Boland CR Goel A. Microsatellite instability in colorectal cancer. Gastroenterology (2010) 138:2073–2087.e2073. doi: 10.1053/j.gastro.2009.12.064 20420947 PMC3037515

[24] Chen T Zhang C Liu Y Zhao Y Lin D Hu Y. A gastric cancer LncRNAs model for MSI and survival prediction based on support vector machine. BMC Genomics (2019) 20:846. doi: 10.1186/s12864-019-6135-x 31722674 PMC6854775

[25] Smyth EC Wotherspoon A Peckitt C Gonzalez D Hulkki-Wilson S Eltahir Z. Mismatch Repair Deficiency, Microsatellite Instability, and Survival: An Exploratory Analysis of the Medical Research Council Adjuvant Gastric Infusional Chemotherapy (MAGIC) Trial. JAMA Oncol (2017) 3:1197–203. doi: 10.1001/jamaoncol.2016.6762 PMC5824280 28241187

[26] Yeo SA Chew MH Koh PK Tang CL. Young colorectal carcinoma patients do not have a poorer prognosis: a comparative review of 2,426 cases. Techniques coloproctology (2013) 17:653–61. 10.1007/s10151-013-0977-z 23460362

[27] Zhao L Bao F Yan J Liu H Li T Chen H. Poor prognosis of young patients with colorectal cancer: a retrospective study. Int J Colorectal Dis (2017) 32:1147–56. 10.1007/s00384-017-2809-5 28389779

[28] Okamoto K Sasaki K Nozawa H Murono K Emoto S Yamauchi S. Poor prognosis of young male patients with stage III colorectal cancer: A multicenter retrospective study. J Surg Oncol (2023). 10.1002/jso.27557 38115553

[29] André T Shiu KK Kim TW Jensen BV Jensen LH Punt C. Pembrolizumab in Microsatellite-Instability-High Advanced Colorectal Cancer. New Engl J Med (2020) 383:2207–18. 10.1056/NEJMoa2017699 33264544

[30] Leão B Wen X Duarte HO Gullo I Gonçalves G Pontes P. Expression of Thomsen-Friedenreich Antigen in Colorectal Cancer and Association with Microsatellite Instability. Int J Mol Sci (2021) 22:1340. 33572915 10.3390/ijms22031340 PMC7866256

[31] Argilés G Tabernero J Labianca R Hochhauser D Salazar R Iveson T. Localised colon cancer: ESMO Clinical Practice Guidelines for diagnosis. Treat follow-up Ann Oncol (2020) 31:1291–305. 10.1016/j.annonc.2020.06.022 32702383

[32] Mouradov D Domingo E Gibbs P Jorissen RN Li S Soo PY. Survival in stage II/III colorectal cancer is independently predicted by chromosomal and microsatellite instability, but not by specific driver mutations. Am J Gastroenterol (2013) 108:1785–93. 10.1038/ajg.2013.292 24042191

[33] Guastadisegni C Colafranceschi M Ottini L Dogliotti E. Microsatellite instability as a marker of prognosis and response to therapy: a meta-analysis of colorectal cancer survival data. Eur J Cancer (Oxford Engl 1990) (2010) 46:2788–98. 10.1016/j.ejca.2010.05.009 20627535

[34] Alwers E Jansen L Bläker H Kloor M Tagscherer KE Roth W. Microsatellite instability and survival after adjuvant chemotherapy among stage II and III colon cancer patients: results from a population-based study. Mol Oncol (2020) 14:363–72. 10.1002/1878-0261.12611 PMC6998383 31816156

[35] Morse MA Hochster H Benson A. Perspectives on Treatment of Metastatic Colorectal Cancer with Immune Checkpoint Inhibitor Therapy. Oncologist (2020) 25:33–45. 31383813 10.1634/theoncologist.2019-0176 PMC6964145

[36] Lichtenstern CR Ngu RK Shalapour S Karin M. Immunotherapy, Inflammation and Colorectal Cancer, Cells, 9. (2020). doi: 10.3390/cells9030618 PMC7140520 32143413

[37] Oliveira AF Bretes L Furtado I. Review of PD-1/PD-L1 Inhibitors in Metastatic dMMR/MSI-H Colorectal Cancer. Front Oncol (2019) 9:396. doi: 10.3389/fonc.2019.00396 31139574 PMC6527887

[38] Toh JWT Phan K Reza F Chapuis P Spring KJ. Rate of dissemination and prognosis in early and advanced stage colorectal cancer based on microsatellite instability status: systematic review and meta-analysis. Int J Colorectal Dis (2021) 36:1573–96. doi: 10.1007/s00384-021-03874-1 33604737

[39] Kang S Na Y Joung SY Lee SI Oh SC Min BW. The significance of microsatellite instability in colorectal cancer after controlling for clinicopathological factors. Med (Baltimore) (2018) 97:e0019. doi: 10.1097/MD.0000000000010019 PMC5851768 29489646

[40] Kim JW Shin MK Kim BC. Clinicopathologic impacts of poorly differentiated cluster-based grading system in colorectal carcinoma. J Korean Med Sci (2015) 30:16–23. doi: 10.3346/jkms.2015.30.1.16 25552879 PMC4278023

[41] Nazemalhosseini-Mojarad E Asadzadeh-Aghdaei H Mohammadpour S Esfahani AT Porhoseingholi MA Molaei M. Downregulation of human leukocyte antigen Class I expression: An independent prognostic factor in colorectal cancer. J Cancer Res Ther (2020) 16:S165–s171. 33380672 10.4103/jcrt.JCRT_429_18

[42] Wang HC Chou CL Yang CC Huang WL Hsu YC Luo CW. Over-Expression of CHD4 Is an Independent Biomarker of Poor Prognosis in Patients with Rectal Cancers Receiving Concurrent Chemoradiotherapy. Int J Mol Sci 20 (2019). doi: 10.3390/ijms20174087 PMC6747537 31438571

