# Supplementary material for: Correlation between mismatch repair statuses and the prognosis of stage I–IV colorectal cancer
Source: Front Oncol. 2024 Jan 29;13:1278398. doi: 10.3389/fonc.2023.1278398 (PMC10859923; doi:10.3389/fonc.2023.1278398)
Supplement: Supplementary file 1 [file Table_1.docx]

Supplementary Table 1 comparison of clinic and pathologic features between MSI-H and MSI-L&MSS(N,%,M±SD)

| Factors | MSI-H(n=339) | MSI-L&MSS(n=1418) | P |
| --- | --- | --- | --- |
| Sex |  |  | 0.968 |
| Male | 171（19.3） | 717（80.7） |  |
| Female | 168（19.3） | 701（80.7） |  |
| ASA |  |  | 0.794 |
| 1 | 255（19.6） | 1044（80.4） |  |
| 2 | 74（18.1） | 334（81.9） |  |
| 3 | 10（20.0） | 40（80.0） |  |
| Tumor location |  |  | 0.006* |
| Ileocecum | 33（22.4） | 114（77.6） |  |
| Right colon | 24（14.0） | 148（86.0） |  |
| Transverse colon | 63（22.7） | 214（77.3） |  |
| Left colon | 42（13.0） | 282（87.0） |  |
| Sigmoid colon | 36（21.6） | 131（78.4） |  |
| Rectum | 141（21.0） | 529（79.0） |  |
| Operation method |  |  | 0.151 |
| RHC | 71（18.0） | 323（82.0） |  |
| LHC | 113（17.5） | 531（82.5） |  |
| HO | 13（25.5） | 38（74.5） |  |
| AR | 98（19.9） | 394（80.1） |  |
| APR | 44（25.0） | 132（75.0） |  |
| Pt status |  |  | 0.000a** |
| Tis | 3（18.8） | 13（81.2） |  |
| T1 | 35（31.0） | 78（69.0） |  |
| T2 | 46（14.6） | 268（85.4） |  |
| T3 | 91（14.4） | 539（85.6） |  |
| T4a | 58（20.8） | 221（79.2） |  |
| T4b | 106（26.2） | 299（73.8） |  |
| Differentiation |  |  | 0.000** |
| Well | 59（26.2） | 166（73.8） |  |
| Moderate | 227（20.0） | 909（80.0） |  |
| Poor or Non | 53（13.4） | 343（86.6） |  |
| Complication |  |  | 0.742 |
| No | 313（19.4） | 1299（80.6） |  |
| Yes | 26（17.9） | 119（82.1） |  |
| Chemotherapy |  |  | 0.000** |
| Yes | 275（17.6） | 1285（82.4） |  |
| No | 64（32.5） | 133（67.5） |  |
| N Stage |  |  | 0.003* |
| N0 | 122（24.3） | 380（75.7） |  |
| N1 | 118（17.9） | 541（82.1） |  |
| N2 | 99（16.6） | 497（83.4） |  |
| AJCC-8 |  |  | 0.000** |
| Ⅰ | 52（35.4） | 95（64.6） |  |
| Ⅱ | 54（24.0） | 171（76.0） |  |
| Ⅲ | 154（17.6） | 721（82.4） |  |
| Ⅳ | 79(15.5) | 431(84.5) |  |
| Age | 63.9±13.9 | 63.6±13.9 | 0.725 |
| Tumor size （cm） | 3.7±1.1 | 3.8±1.0 | 0.040* |
| Operation time(min) | 1503.±32.8 | 150.8±32.1 | 0.812 |
| Resection length(cm) | 27.4±10.4 | 28.2±10.0 | 0.194 |
| Blood loss (ml) | 194.7±86.1 | 191.3±94.2 | 0.546 |
| Preoperative S-CEA | 1.57±0.5 | 1.50±0.5 | 0.027* |
| Lymph harvest (n) | 14.2±1.7 | 14.1±1.9 | 0.662 |
| Positive lymph (n) | 2.62±2.8 | 2.88±2.7 | 0.041* |

RHC: Right hemicolectomy, LHC: Left hemicolectomy, HO: Hartmann operation, AR: Anterior resection, APR: Abdominal perineal resection, ASA: American Society of Anesthesiologists ;Pt : Pathologic tumor , a：Fisher Exact test；*P<0.05,**P<0.001
