# Supplementary material for: Correlation between mismatch repair statuses and the prognosis of stage I–IV colorectal cancer
Source: Front Oncol. 2024 Jan 29;13:1278398. doi: 10.3389/fonc.2023.1278398 (PMC10859923; doi:10.3389/fonc.2023.1278398)
Supplement: Supplementary file 2 [file Table_2.docx]

Supplementary Table 2 Correlation analyses between MSI and Clinicopathologic features

| MSI | Spearman’s rho | P |
| --- | --- | --- |
| Preoperative S-CEA | -0.053 | 0.028* |
| Positive lymph | 0.050 | 0.037* |
| Tumor location | -0.022 | 0.355 |
| Tumor size | 0.056 | 0.019* |
| p T status | -0.065 | 0.007* |
| N stage | 0.074 | 0.002* |
| Differentiation | 0.096 | 0.000** |
| Chemotherapy | -0.119 | 0.000** |
| AJCC-8 | 0.113 | 0.000** |

*P<0.05,**P<0.001
