# Supplementary material for: Correlation between mismatch repair statuses and the prognosis of stage I–IV colorectal cancer
Source: Front Oncol. 2024 Jan 29;13:1278398. doi: 10.3389/fonc.2023.1278398 (PMC10859923; doi:10.3389/fonc.2023.1278398)
Supplement: Supplementary file 3 [file Table_3.docx]

Supplementary Table 3 Univariate and multivariate analysis of prognosis for colorectal cancer

| Univariate analysis | | | | | | Multivariate analysis | | |
| --- | --- | --- | --- | --- | --- | --- | --- | --- |
| Factor | N | Harzard Ratio(HR) | 95%CI for survival time(60months) | 5-year OS(%) | P | HR (95%CI) | Wald | P |
| Sex |  |  |  |  | 0.303 |  |  | N/A |
| male | 888 | Ref. | 42.20~44.77 | 47.4 |  |  |  |  |
| female | 869 | 1.071 | 43.04~45.64 | 49.9 |  |  |  |  |
| Age (years) |  |  |  |  | <0.001** |  |  | 0.610 |
| ≤60 | 533 | Ref. | 46.54~49.48 | 55.0 |  | Ref. |  |  |
| ＞60 | 1224 | 1.440 | 40.99~43.25 | 45.9 |  | 1.041(0.893~1.213) | 0.260 |  |
| Size (cm) |  |  |  |  | <0.001** |  |  | 0.044* |
| ≤2 | 84 | Ref. | 58.85~59.87 | 91.7 |  | Ref. |  |  |
| ＞2&≤5 | 1466 | 8.58 | 42.84~44.84 | 47.7 |  | 2.159(0.974~4.786) | 3.589 | 0.058 |
| ＞5 | 207 | 11.69 | 35.37~40.92 | 37.7 |  | 2.570(1.131~5.841) | 5.078 | 0.024* |
| P T status |  |  |  |  | <0.001** |  |  | <0.001** |
| Tis | 16 | Ref. | 57.21~60.41 | 87.5 |  | Ref. |  |  |
| T1 | 113 | 1.586 | 56.74~58.90 | 80.4 |  | 0.097(0.015~0.619) | 6.082 | 0.014* |
| T2 | 314 | 5.894 | 50.85~52.18 | 46.4 |  | 0.151(0.024~0.956) | 4.031 | 0.045* |
| T3 | 630 | 7.136 | 39.77~42.89 | 38.3 |  | 0.234(0.037~1.490) | 2.365 | 0.035* |
| T4a | 279 | 2.553 | 45.21~48.38 | 37.5 |  | 0.144(0.022~0.925) | 4.172 | 0.041* |
| T4b | 405 | 7.524 | 35.68~39.77 | 35.6 |  | 0.150(0.023~0.995) | 4.034 | 0.045* |
| Differentiation |  |  |  |  | <0.001** |  |  | <0.001** |
| Well | 225 | Ref. | 51.24~55.15 | 74.6 |  | Ref. |  |  |
| Moderate | 1136 | 2.031 | 45.02~47.25 | 55.9 |  | 1.531(1.150~2.038) | 8.509 | 0.004* |
| Poor&Non | 396 | 5.907 | 30.46~33.99 | 12.9 |  | 3.862(2.851~5.231) | 76.143 | <0.001** |
| N Stage |  |  |  |  | <0.001** |  |  | <0.001** |
| N0 | 502 | Ref. | 55.11~56.87 | 73.4 |  | Ref. |  |  |
| N1 | 659 | 1.639 | 47.32~49.98 | 62.5 |  | 0.500(0.376~0.664) | 22.823 | <0.001** |
| N2 | 396 | 7.473 | 27.03~29.92 | 12.4 |  | 1.021(0.767~1.358) | 20.221 | <0.001** |
| AJCC-8 |  |  |  |  | <0.001** |  |  | <0.001** |
| Ⅰ | 147 | Ref. | 59.26~59.96 | 96.6 |  | Ref. |  |  |
| Ⅱ | 225 | 7.082 | 58.14~59.00 | 76.6 |  | 5.361(1.647~17.45) | 7.777 | 0.005* |
| Ⅲ | 875 | 14.22 | 51.08~52.88 | 60.9 |  | 12.781(3.973~41.118) | 18.266 | <0.001** |
| Ⅳ | 510 | 200.84 | 18.14~19.87 | 1.0 |  | 257.588(79.310~836.613) | 85.311 | <0.001** |
| MSI |  |  |  |  | <0.001** |  |  | <0.001** |
| MSI-H | 339 | Ref. | 50.14~53.05 | 63.3 |  | Ref. |  |  |
| MSI-L&MSS | 1418 | 1.842 | 41.01~43.13 | 45.1 |  | 3.328(2.701~4.099) | 127.780 |  |

*P<0/05; **P<0.001; N/A: not performed because P>0.05in univariate analysis.
